# Effect of education program regarding pathological jaundice on nurses’ performance and neonates’ bilirubin-induced neurological dysfunction

**DOI:** 10.1007/s00431-024-05921-9

**Published:** 2025-01-07

**Authors:** Nora Abd El-Alim Ebrahim, Madiha Hassan Bayoumi, Hanan El-Sayed Metwally

**Affiliations:** Pediatric Nursing, Faculty of Nursing Affiliated to Benha University, Benha, Egypt

**Keywords:** Education program, Bilisphere phototherapy, Nurses’ performance, Outcomes of neonates, Pathological jaundice

## Abstract

Both term and preterm infants are susceptible to pathological jaundice, a frequent condition that can cause long-lasting neurological damage. A novel treatment for indirect pathological hyperbilirubinemia is bilisphere phototherapy, which lowers total serum bilirubin just as well as exchange transfusions. A quasi-experimental research design was utilized in the current study. This study was conducted in Neonatal Intensive Care Unit at Benha Specialized Pediatric Hospital. A convenient sample of all available nurses (60) working in the previously mentioned setting. A purposive sample of neonates (90) with pathological jaundice which would be divided into control and study groups. Three tools were used: Tool I: A structured interview questionnaire sheet, Tool II: observational checklist for caring neonates with pathological jaundice, and Tool III: neonatal outcomes assessment sheet. There was a positive correlation between nurses’ total performance regarding pathological jaundice and bilisphere phototherapy at pre-/post-education program implementation. Additionally, there were a positive correlation between neonates’ outcomes in the study group and nurses’ total performance post-program implementation.

Conclusion the education program regarding pathological jaundice was effective in improving nurses’ performance and neonates’ bilirubin-induced neurological dysfunction post compared to pre-education program implementation.

**Table:** 

**What is Known:** • *Pathological jaundice had directly effect on neurological status in neonates as aresults of accumulated bilirubin in basal gangelia in brain and bilirubin induced neurological dysfunction score considered important tool to indicate acute bilirubin encephalopathy.*
**What is New:** • *Education programs and periodic training to medical team provide improvement their performance, nurse had significant role to provide neonates care in NICU. So, improve nurses performance lead to improve neonate outcomes.* • *Bilisphere phototherapy is an important device which had positive outcomes in pediatrics which improve neonates’ health status, but its effect depends on provided effective nursing practice during therapy.* • *Pathological jaundice management with bilisphere phototherapy and bilirubin induced neurological dysfunction score working together to provide optimal care for neonates, which bilisphere phototherapy decrease bilirubin level and BIND score assess neurological status and detect any abnormalites during therapy.*

**What is Known:**

• *Pathological jaundice had directly effect on neurological status in neonates as aresults of accumulated bilirubin in basal gangelia in brain and bilirubin induced neurological dysfunction score considered important tool to indicate acute bilirubin encephalopathy.*

**What is New:**

• *Education programs and periodic training to medical team provide improvement their performance, nurse had significant role to provide neonates care in NICU. So, improve nurses performance lead to improve neonate outcomes.*

• *Bilisphere phototherapy is an important device which had positive outcomes in pediatrics which improve neonates’ health status, but its effect depends on provided effective nursing practice during therapy.*

• *Pathological jaundice management with bilisphere phototherapy and bilirubin induced neurological dysfunction score working together to provide optimal care for neonates, which bilisphere phototherapy decrease bilirubin level and BIND score assess neurological status and detect any abnormalites during therapy.*

## Introduction

The yellowish discoloration of the skin and sclera in a neonate is known as neonatal jaundice or hyperbilirubinemia. Unlike adults, jaundice appears when total bilirubin exceeds 2 mg/dL, and yellowish discoloration appears when serum bilirubin exceeds 4–5 mg/dL in a neonate. The spread of discoloration is cephalocaudal, as initially jaundice appears on the face and then spreads to the extremities in relation to increase in bilirubin level [1]. Worldwide, neonatal jaundice (NJ) affects 1.1 million neonates every year [2].

Pathological hyperbilirubinemia includes jaundice appearing within the 1st day of life or a rate of serum bilirubin level exceeding 0.2 mg/dL/h. The most common causes of pathological jaundice are hemolytic disease: e.g., RH or ABO incompatibility and infection: congenital (e.g., toxoplasmosis, rubella) or postnatal infection [3].

While neonatal jaundice is typically temporary and goes away by the end of the first week after birth, severe hyperbilirubinemia can cause acute bilirubin encephalopathy and kernicterus, which can be fatal in the first few months of life. Neonates who survive this condition frequently experience mental retardation, movement and balance issues, seizures, high-frequency hearing loss, and speech impairment. Therefore, prompt diagnosis and treatment of neonatal jaundice is crucial to avoid more complications [4].

Pathologic jaundice management includes maintaining hydration, and phototherapy and medications such as intravenous immunoglobulin (IVIG) infusion and exchange transfusion are considered for treatment of severe pathological jaundice. Phototherapy is the use of a strong light source to change the bilirubin in the blood to a water-soluble form that can be excreted. The light source needs to emit more than 30 mw/cm^2^/nm to be effective [5].

The intensity of the phototherapy depends on several factors: Type and number of light tubes, area covered by the lights, and distance of the neonate from the light. Conventional phototherapy delivers an intensity of light in the range of 5–10 mw/cm^2^/nm, while bilisphere phototherapy delivers an intensity of > 30 mw/cm^2^/nm. Bilisphere phototherapy with lights deliver a double dose of light from above and below which is usually very effective in decreasing the bilirubin level [6]. Bilisphere phototherapy decreases bilirubin by 30–40% within 24 h and up to 10 mg/dL in the first 6 h when total serum bilirubin (TSB) levels are more than 30 mg/dL [7].

Nursing care for pathological jaundice consists of observing the neonate’s skin, sclera, and mucous membranes (blanching the skin over bony prominences enhances the evaluation for jaundice). Maintain fluid intake by increasing the frequency of feeding or through intravenous (IV) therapy. The neonates need special eye protectors as the bilisphere phototherapy lights may cause retinal damage; regularly checked for any discharge. The neonate’s diapers were checked, and the buttocks were monitored for any skin breakdown due to an increase in stooling related to bilisphere phototherapy [3].

In order to preserve the lives of newborns, neonatal nurses need to learn cutting-edge, modern techniques like phototherapy and blood exchange. Nurses have a crucial role in obtaining optimal outcomes for infants who are suffering from pathological jaundice. The current study was carried out to shed light on nurses’ knowledge and practice related care of neonates suffering from pathological jaundice under bilisphere phototherapy [8].

### Significance of the study

The rate of severe infant hyperbilirubinemia was 4.4/10,000 in the USA, whereas it was the highest in the African region (667.8) per 10,000 live births. According to the Bhutani nomogram, 1.9% of 4000 healthy full-term neonates who were examined for jaundice had bilirubin levels in the high-risk zone, while the precise figures are unclear in Egypt. In a population with 2.5 million births every year, this suggests that a significant section of the population may be at risk for severe hyperbilirubinemia and its consequences [9].

The corresponding mortality rates in the Eastern Mediterranean, Africa, South-East Asia, and Europe were 13.02%, 7.52%, 2.01%, and 0.07% due to pathological jaundice [10]. One in 10,000 live births is thought to be affected by acute bilirubin encephalopathy, while 1 in 50,000 to 100,000 live births is thought to be affected by chronic bilirubin encephalopathy [11].

Numerous Egyptian studies have documented a high number of neonates with bilirubin encephalopathy, despite the absence of a national registry for kernicterus cases in Egypt. In Egyptian children, kernicterus accounted for 9% of all etiologies and was the third most common cause of developmental delay. According to other research, one of the main causes of cerebral palsy in Egyptian children was neonatal jaundice. The majority of severe hyperbilirubinemia and kernicterus cases worldwide are said to occur in Egypt and other middle- and low-income nations [12]. Neurological impairment is likely to be avoided with early detection and treatment with bilisphere phototherapy and exchange transfusion [13].

### Aim of the study

This study’s aim is to evaluate the nurse training curriculum for intensive care management (including phototherapy) on their knowledge and skill performance including the bilirubin-induced neurological dysfunction score and well-being of mother–child dyad prior to hospital discharge.

### Research hypotheses

A Nurses’ knowledge and skill performance will improve with a targeted training program for both specific neonatal assessment of hyperbilirubinemia and use of phototherapy.
B Use of BIND scores for severe hyperbilirubinemia neonates will improve with phototherapy device for trained nurses.
C The quality of training (as measured in study of “Hypothesis A”) will correlate to BIND score outcomes (as measured in “Hypothesis B”).

## Methods

### Research design

A quasi-experimental research design was utilized to conduct the study.

### Setting

The current study was conducted in Neonatal Intensive Care Unit at Benha Specialized Pediatric Hospital which affiliated to Secretariat of Specialized Medical Centers, at Benha city. The Neonatal Intensive Care Unit is located on the 3rd and 5th floor of the medical building, the 3rd floor consists of 1 room with 28 incubators, and the 5th floor consists of 1 room with 7 incubators. The total number of bilisphere phototherapy are three devices.

### Sample

This study included the following:

- A convenient sample of all available nurses (60) working in the previously mentioned setting
- A purposive sample of neonates (90) with pathological jaundice which will be divided into control (45) and study (45) groups under the following inclusion criteria:

Inclusion criteria of neonates are as follows:

1. Neonates with gestational age 35–42 weeks, according to [14]
2. Neonates with indirect hemolytic hyperbilirubinemia.
3. Neonatal age not exceed 7 days.
4. Neonates under bilisphere phototherapy.

Exclusion criteria of neonates are as follows:

1. Neonates with acute bilirubin encephalopathy or kernicterus.
2. Neonates suffering from any life-threatening conditions or congenital malformations, such as heart defects and neural tube defects.
3. Neonates with neurological disorders, such as seizures and epilepsy.
4. Preterm neonates under 35 weeks of gestational age.

### Tools for data collection

Three tools were utilized to achieve the aim of the current study:

#### Tool (I): A structured interview questionnaire sheet

This tool was designed, adjusted, and prepared in Arabic language by the researcher and reviewed by the supervisors to collect data about characteristics of the nurses and the neonates after reviewing related literatures. It consisted of four parts:

o *Part 1: Nurses’ characteristics included the following*: Age, gender, level of education, experience years, and training courses regarding bilisphere phototherapy
o *Part 2: Neonates’ characteristics as follows*: Age, gestational age, gender, birth weight, type of feeding and medical history as medical cause of pathological jaundice, bilirubin level during use bilisphere phototherapy, mother blood group, neonate blood group, mother RH, mother RH, neonate RH, and parents blood relation.
o *Part 3: Nurses’ knowledge regarding neonatal pathological jaundice*: It was used to assess nurse’s knowledge regarding pathological jaundice, based on [15–18] and modified by the researcher; it included *11 questions* about definition of bilirubin, types of bilirubin, neonate’s normal bilirubin level, definition, causes, signs and symptoms, risk factors, diagnosis methods, complications, preventive methods, and management methods of neonates’ pathological jaundice. The total score of 11 questions were 22°.
o *Part 4: Nurses’ knowledge regarding bilisphere phototherapy:* It was used to assess nurse’s knowledge regarding bilisphere phototherapy, based on [19–21] and modified by the researcher; it included *10 questions* about definition, components, indications, contraindications, advantages, duration of one cycle of bilisphere phototherapy, the bilirubin level requiring use of bilisphere phototherapy, the range of bilirubin breakdown by bilisphere phototherapy, the nursing care during bilisphere phototherapy, and the complications of bilisphere phototherapy. The total score of 10 questions were 20°.

### *Scoring system of nurses’ knowledge*

Scoring system for knowledge of the studied nurses would be categorized as the following: The studied nurses’ answers would be compared with a model key answer, and 2 scores would be given for complete and correct answer, 1 score for incomplete correct answer, and 0 score for incorrect answer or do not know.

The total scores of nurses’ knowledge would be calculated as the following:

The total score of knowledge items were 42°.

o *If the nurses’ answers* ≥ 36°, which represents ≥ 85%, it is considered satisfactory knowledge.
o *If the nurses’ answers* < 36°, which represents < 85%, it is considered unsatisfactory knowledge.

#### Tool (II): Observational checklist for caring neonates with pathological jaundice under bilisphere phototherapy

It was used to assess nurses’ practice regarding bilisphere phototherapy adapted from [20, 22, 23]and modified by the researcher; it consisted of three parts as the following:

- *Part 1*: Nurses’ actual practice before bilisphere phototherapy consists of *46 items*, which consist of 2 parts: neonate preparation and bilisphere preparation which include assess air and skin probes, assess function of lambs and height, and adjust bilisphere atmosphere. The total score is 46°.
- *Part 2*: Nurses’ actual practice during bilisphere phototherapy consists of *25 items*, and the total score are 25°.
- *Part 3*: Nurses’ actual practice after bilisphere phototherapy consists of *21 items*, which consist of 3 parts: assess neonate condition, cleaning and disinfection of the equipment, and cleaning the hammock. The total score is 21°.

### Scoring system of nurses’ practice

The scoring system for practice of the studied nurses would be calculated as the following: The nurses’ practice would be categorized into done (1) score and not done (0) score.

The total scores of nurses’ practice would be calculated as the following:

The total score of practice items were 92°.

o *If the nurses’ answers* ≥ 78°, which represents = 85–100%, it is considered competent practice.
o *If the nurses’ answers* < 78°, which represents < 85%, it is considered incompetent practice.

#### Tool (III): Neonatal outcomes assessment sheet

It is collected by researcher immediately after discharge from bilisphere phototherapy to ensure all neonate conditions have effect of bilisphere whether positive, which neonate has stability condition or negative effect, and which neonate needs more interference. It consisted of two parts:

- *Part 1* **—*** Neonatal medical assessment sheet*: It was modified by the researcher based on [24–27]. It include data about the neonate and would be extracted from the neonate’s medical assessment sheet such as vital signs, intended range of laboratory investigations (is range which the neonate under bilisphere phototherapy hopeful to achieve to continue under conventional phototherapy, but unless not achieve, the neonate has many options according to condition as follows: repeat another cycle of bilisphere phototherapy and adding medication with bilisphere phototherapy or exchange transfusion), feeding patterns, skin characteristics, urine characteristics, stool characteristics, side effects of bilisphere phototherapy, exchange transfusion, and health outcomes of neonates immediately after discharge from bilisphere phototherapy.
- *Part 2 — Bilirubin-induced neurological dysfunction (BIND)*: It was adopted from [28]. After receiving acceptance and the permission of *Dr. Vinod Bhutani*, the corresponding author of the original BIND scoring, the researcher assessed neonatal neurological status and evaluated severity of pathological jaundice, and it would include mental status, muscle tone, and cry pattern. The BIND score was published several times in Elsevier publisher with Dr. Vinod et al. and the modified BIND score published in Springer publisher.

### Scoring system of BIND

BIND score = sum (points for all three parameters). The total score would be categorized as follows:

1. The score 7 to 9 represents severe acute bilirubin encephalopathy; urgent interventions are recommended to possibly minimize further brain injury.
2. The score 4 to 6 represents moderate acute bilirubin encephalopathy and is likely to be reversible with urgent bilirubin reduction.
3. The score 1 to 3 represents mild acute bilirubin encephalopathy and is usually reversible with urgent bilirubin reduction strategies.
4. The score of zero means no indication of acute bilirubin encephalopathy.

### Pilot study

In order to assess the validity and suitability of the research instruments and determine how long nurses should take to complete the questionnaire, a pilot study comprising 10% of the sample size—six nurses and four neonates with pathological jaundice receiving bilisphere phototherapy was conducted in September 2023 for 1 month. Since the pilot research showed that no significant changes were made to the study instruments, all participants were included in the sample.

### Content validity

A panel of three pediatric nursing professors from Benha University’s Faculty of Nursing reviewed the data collection tools to assess how well the items related to one another, as well as their clarity, relevance, comprehensiveness, simplicity, and applicability. Reliability was then conducted to confirm the validity of the study tools, including adding questions about pathological jaundice risk factors and dividing nurses’ actual practice before, during, and after bilisphere phototherapy.

### Reliability

Reliability of the study tools was checked by testing its internal consistency using Cronbach’s alpha coefficient test.

- Knowledge questionnaire reliability statistics Cronbach’s alpha = 0.960
- Observational checklist reliability statistics Cronbach’s alpha = 0.988
- Neurological scale reliability statistics Cronbach’s alpha = 0.998

This indicated a high degree of reliability for the study tools.

### Field work

To accomplish the current study’s goal, the following phases were chosen: assessment, planning, implementation, and evaluation. These phases span 8 months, starting in early October 2023 and ending in late May 2024. It was gathered in compliance with the study setting’s guidelines. In the Neonatal Intensive Care Unit, data was gathered 3 days a week (Sunday, Tuesday, and Thursday) during the morning shift from 8 AM to 2 PM and the afternoon shift from 2 to 8 PM.

The researcher divided studied neonates into two groups: the first group is the control group (45) who the researcher assessed before implementing an education program (assessment phase) to assess the effect of nurses’ performance on neonates’ outcomes. The second group is the study group (45) who the researcher assessed after implementing an education program (evaluation phase) to assess the effect of nurses’ performance on neonates’ outcomes.

#### (a) Assessment phase

Data was collected in this phase before implementing the education program. The questionnaire sheets were distributed to all nurses individually to assess nurse’s performance and determine nurses’ needs regarding pathological jaundice and bilisphere phototherapy using the previous study tools.

The average time needed to answer personal data and knowledge questions is 10–15 min. Each nurse was observed individually during their actual practice without observation from nurses to evaluate their practice exactly using an observational checklist, and the average time needed for the completion of observational checklist for each nurse (by the researcher) ranged between 20 and 30 min. The average number of nurses interviewed per day was 2–3 nurses per day.

Also, the data of the studied neonates (control group) which was collected by the researcher ranged between 10 and 15 min. The time needed for filling out all data collection tools was 40–60 min.

#### (b) Planning phase

This stage involved determining the true needs of the nurses under study and analyzing the results of the assessment phase (pretest). In order to help nurses grasp the teaching program, the researcher created a booklet with easy Arabic text and illustrations.

#### (c) Implementation phase

After evaluating the nurses’ performance and identifying their requirements for bilisphere phototherapy and pathological jaundice, it was put into effect. Eight sessions, three for the theoretical portion and five for the practical portion over 3 days per week in the NICU, were required to accomplish this. Every session began with an overview of the one before it and the current session’s objective. Throughout the session, nurses and the researcher sit in a circle and alternate sharing; each nurse has a chance to give information and ask any questions.

Eight sessions in all, lasting 45–60 min each, were conducted over the course of 4 months (from December 2023 to the end of March 2024). Additionally, eight sessions with the study objectives were conducted using various media and instructional techniques.

A schedule including the date, time, location, subjects, and length of each session was created that is appropriate for nurses. The workload in the NICU made it difficult to take all the nurses at once. In order to take precautions, they split up into 12 groups of 4 to 6 nurses per session. A copy of the teaching software and the movie were sent to each nurse’s mobile device, or they were given a copy on CD. In order to facilitate motivation, communication, interaction, support, and follow-up, researchers created a WhatsApp group and added nurses.

*Precautionary measures* are taken into consideration during data collection and sessions including the following:

- Personnel protective materials such as a face mask, gloves, and antiseptic solution for hand hygiene
- Personal distancing to maintain a minimum 1.5-m distance
- Avoiding shaking hands or hugging
- Always cover the mouth while sneezing and coughing to prevent droplet transmission
- Avoid touching one’s mouth, nose, or eyes to prevent the spread of infection

#### (d) Evaluation phase

After implementation of education program regarding bilisphere phototherapy, the posttest was administered to assess nurse’s knowledge and practice; also, the researcher assessed outcomes of the studied neonates (study group) using the same tools of the pretest. This helped to evaluate the effect of the implemented education program on improving nurses’ performance and outcomes of neonates with pathological jaundice. The average number of nurses interviewed per day was 2–3 nurses per day, and the period of posttest took 2 months (from the beginning of April 2024 to the end of May 2024).

### Administrative design

After submitting a letter from the dean of Benha University’s Faculty of Nursing, the managers of the mentioned setting formally granted authorization for data collection to be conducted. A thorough explanation of the study’s purpose, significance, and anticipated results were provided.

### Statistical analysis

To make it appropriate for computer entry, the gathered data was coded and converted into a specially created form. SPSS (Statistical Package of Social Science) version 20 was used to enter and evaluate the data. Version 2010 of Microsoft Office Excel was used to create the software visuals. The mean and standard deviation were used to display the quantitative data. The chi-square (*χ*^2^) test was used to examine the qualitative data, which was displayed as frequency distribution tables, numbers, and percentages.

## Results

Table 1 shows that less than half (48.3%) of the studied nurses are in the age group 30 < 40 years. Regarding education level, less than two-thirds (63.3%) of them have technical institute of nursing.

**Table 1:** Percentage distribution of the studied nurses according to their characteristics (*n* = 60)

Nurses’ characteristics	No	%
**Age (years)**		
20 > 30	22	36.7
30 > 40	29	**48.3**
≥ 40	9	15.0
**Mean ± SD**	31.65 ± 5.50	
**Education level**		
Diploma of secondary nursing school	12	20.0
Technical institute of nursing	38	**63.3**
Bachelor in nursing science	10	16.7

Table 2 shows that there is no statistical significant difference between neonates in control and study groups related to age, gender, weight at birth, and type of delivery (*P* > 0.05), while there are a statistical significant difference between neonates in control and study groups related to gestational age (*P* ≤ 0.05).

**Table 2:** Percentage distribution of neonates according to their characteristics (*n* = 90)

**Neonates’ characteristics**	**Control group (*n* = 45)**		**Study group (*n* = 45)**		***χ***^**2**^	***p*-value**
	**No**	**%**	**No**	**%**		
**Age (days)**						
< 1	13	28.9	11	24.4	1.334	0.248 ns
1 < 3	16	35.5	17	37.8		
3 < 5	9	20.0	7	15.6		
5 ≤ 7	7	15.6	10	22.2		
x̅ ± SD	2.42 ± 1.85	2.66 ± 1.97				
**Gestational age (weeks)**						
35 > 37	19	42.2	25	55.6	6.011	0.014*
37 < 39	21	46.7	15	33.3		
39 ≤ 42	5	11.1	5	11.1		
**Gender**						
Girls	28	62.2	26	57.8	0.401	0.527 ns
Boys	17	37.8	19	42.2		
**Weight at birth (grams)**						
2500 > 3000	17	37.8	21	46.7	2.579	0.108 ns
3000 > 3500	18	40.0	16	35.6		
3500 ≤ 3800	10	22.2	8	17.7		
x̅ ± SD	3096.6 ± 390.13	3016.7 ± 387.32				
**Type of delivery**						
Normal vaginal delivery	17	37.8	14	31.1	3.122	0.083 ns
Cesarean section	28	62.2	31	68.9		

^*^A statistical significance at *P* ≤ 0.05

**Fig. 1:**
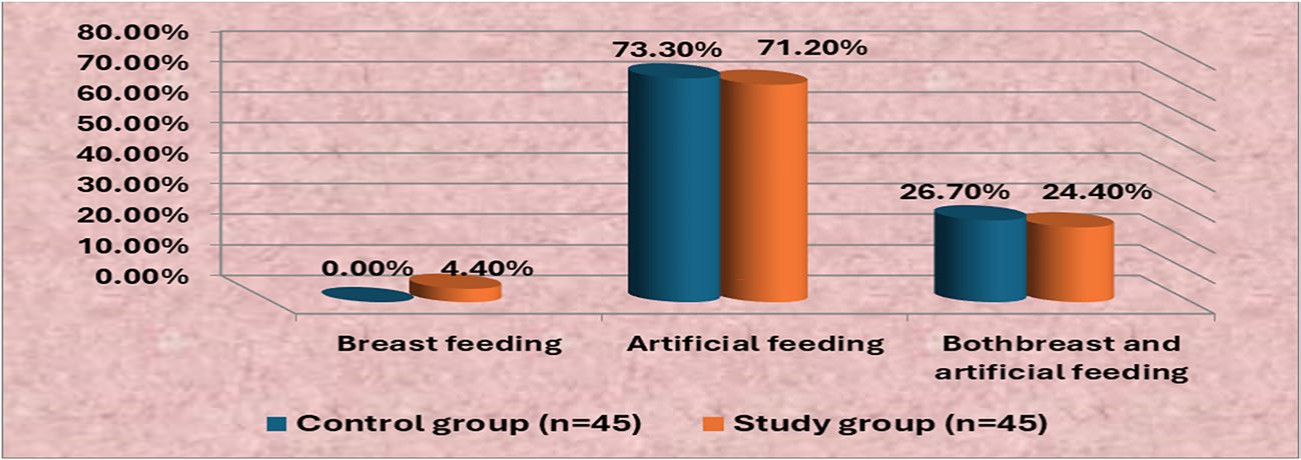
Percentage distribution of the studied neonates in the control and study group according to their type of feeding (*n* = 90)

Figure 1 clarifies that less than three-quarters (73.3% & 71.2%) of the studied neonates in the control and study groups are artificially fed, respectively, while the vast minority (0% & 4.4%) of the control and study groups are breastfed, respectively.

Table 3 reveals that there are no statistical significant differences between neonates in control and study groups according to their medical history (*P* ˃ 0.05).

**Table 3:** Percentage distribution of neonates according to their medical history (*n* = 90)

**Neonate’s medical history**	**Control group (*n* = 45)**		**Study group (*n* = 45)**		***χ***^**2**^	***p*-value**
	**No**	**%**	**No**	**%**		
**Medical causes of pathological jaundice**						
ABO incompatibility	13	28.9	14	31.1	1.511	0.221 ns
Rh incompatibility	16	35.5	17	37.8		
Sepsis	7	15.6	6	13.3		
G6PD	5	11.1	4	8.9		
Thalassemia	4	8.9	4	8.9		
**Bilirubin level on admission (before the use of bilisphere phototherapy) (mg/dl)**						
6 < 10	6	13.3	5	11.1	0.077	0.782 ns
10 < 15	16	35.6	16	35.6		
15 < 20	12	26.7	11	24.4		
≥ 20	11	24.4	13	28.9		
**Mothers blood group**						
A	15	33.3	11	24.4	0.611	0.439 ns
B	16	35.6	19	42.2		
AB	1	2.2	0	0.0		
O	13	28.9	15	33.4		
**Neonates blood group**						
A	19	42.2	16	35.6	0.401	0.527 ns
B	19	42.2	25	55.6		
AB	7	15.6	3	6.7		
O	0	0.0	1	2.1		
**Rh for mother**						
Positive	29	64.5	28	62.2	0.143	0.705 ns
Negative	16	35.5	17	37.8		
**Rh for neonate**						
Positive	45	100.0	45	100.0	0.000	1.0 ns
Negative	0	0.0	0	0.0		
**Is there relation between parents?**						
Yes	26	57.8	21	46.7	2.273	0.132 ns
No	19	42.2	24	53.3		

**Fig. 2:**
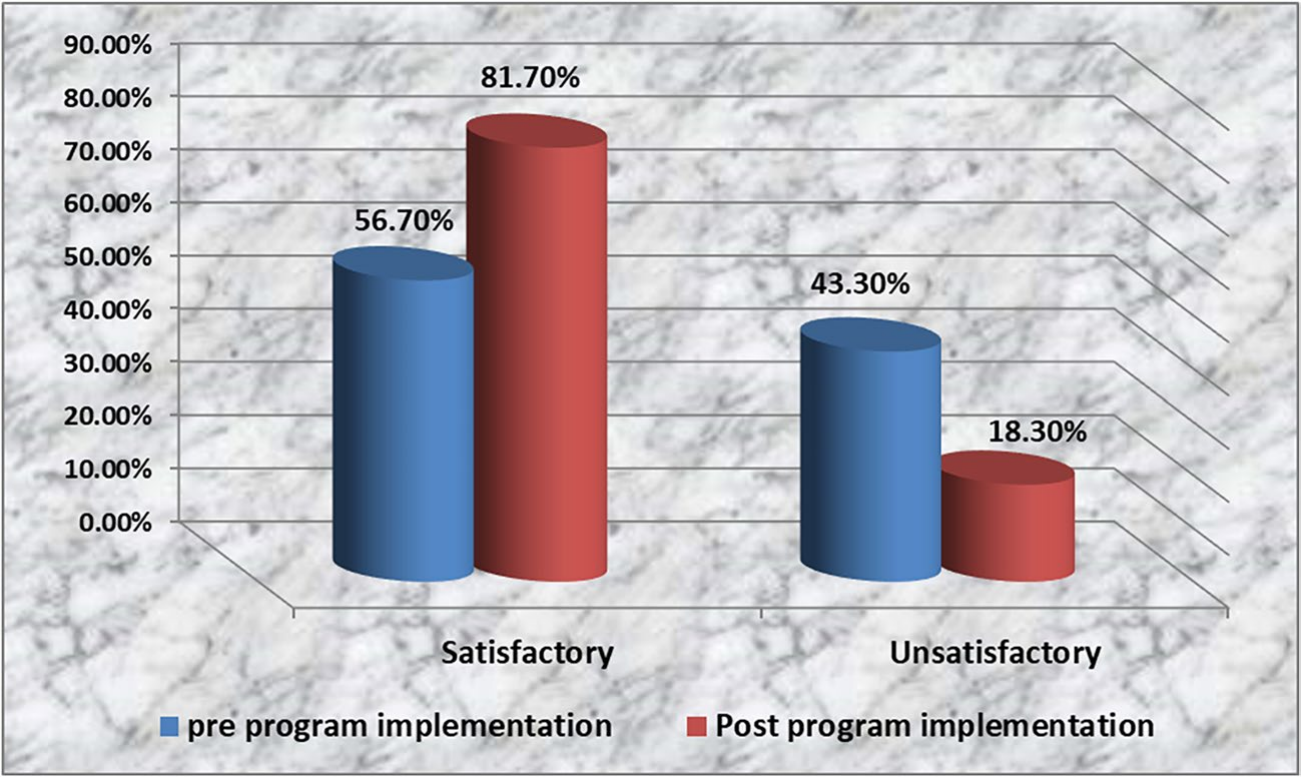
Nurses’ total knowledge regarding pathological jaundice and bilisphere phototherapy pre-/post-education program implementation (*n* = 60)

Figure 2 represents that the majority (81.7%) of the studied nurses have satisfactory knowledge regarding pathological jaundice and bilisphere phototherapy post-education program implementation, compared to more than half of them (56.7%) pre-education program implementation.

**Fig. 3:**
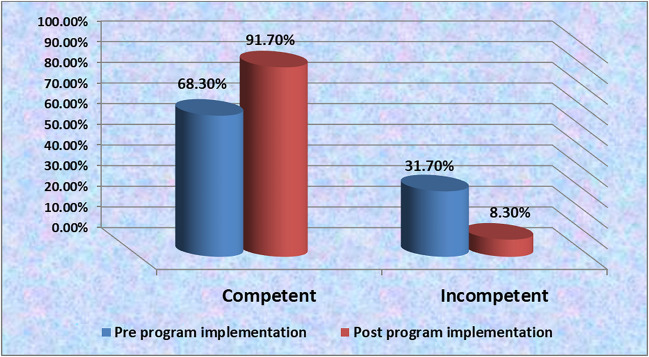
Total score of nurses’ practice regarding care for neonates undergoing bilisphere phototherapy pre-/post-education program implementation (*n* = 60)

Figure 3 portrays that the majority (91.7%) of the studied nurses have competent level of total practice in post-education program implementation compared to more than two-thirds (68.3%) pre-education program implementation.

Table 4 reveals that there are highly statistical significant differences at *P* ≤ 0.001 between the control and study groups regarding all items of their medical assessment.

**Table 4:** Distribution of the studied neonates in the control and study group according to their medical assessment (*n* = 90)

**Neonates’ outcomes**	**Control group (*n* = 45)**				**Study group (*n* = 45)**				***χ***^**2**^	***p*-value**
	**Normal**		**Abnormal**		**Normal**		**Abnormal**			
	**No**	**%**	**No**	**%**	**No**	**%**	**No**	**%**		
**Vital signs**										
Temperature	11	24.4	34	75.6	35	77.8	10	22.2	24.00	0.000**
Pulse	12	26.7	33	73.3	37	61.7	8	13.3	35.00	0.000**
Respiration	18	40.0	27	60.0	38	84.4	7	15.6	28.00	0.000**
Blood pressure	20	44.4	25	55.6	40	88.9	5	11.1	28.00	0.000**
**Intended range of lab investigation**	**Achieved**		**Not achieved**		**Achieved**		**Not achieved**			
Total serum bilirubin test	10	22.2	35	77.8	30	66.7	15	33.3	20.00	0.000**
Hb (hemoglobin)	15	33.3	30	66.7	41	91.1	4	8.9	30.00	0.000**
Reticulocyte	13	28.9	32	71.1	41	91.1	4	8.9	33.00	0.000**
**Feeding pattern**										
Amount	14	31.1	31	68.9	40	88.9	5	11.1	31.00	0.000**
Frequency	17	37.8	28	62.2	42	93.3	3	6.7	31.00	0.000**
**Skin characteristics**										
Skin characteristics	13	28.9	32	71.1	37	82.2	8	17.8	22.154	0.000**
**Urine characteristics**										
Urine characteristics	15	33.3	30	66.7	40	88.9	5	11.1	35.00	0.000**
**Stool characteristics**										
Stool characteristics	11	24.4	34	75.6	40	88.9	5	11.1	37.00	0.000**
**Side effects of bilisphere phototherapy**										
Side effects of bilisphere phototherapy	10	22.2	35	77.8	39	86.7	6	13.3	29.00	0.000**
**Exchange transfusion**										
Exchange transfusion	35	77.8	10	22.2	42	93.3	3	6.7	10.00	0.001**

^**^Highly statistically significant at *P* ≤ 0.001

Table 5 reveals that less than three-quarters (71.1%) of neonates in the study group have no indication of acute bilirubin encephalopathy, compared to less than one-quarter (22.2%) in the control group. Meanwhile, there are highly statistical significant difference at *P* ≤ 0.001 between the control and study group regarding their neurological status and the severity of pathological jaundice.

**Table 5:** Distribution of the studied neonates in the control and study group according to their neurological status and the severity of pathological jaundice (*n* = 90)

**Neonates’ outcomes**	**Control group (*n* = 45)**		**Study group (*n* = 45)**		***χ***^**2**^	***p*-value**
	**No**	**%**	**No**	**%**		
No indication of acute bilirubin encephalopathy	10	**22.2**	32	**71.1**	23.059	0.000**
Mild bilirubin encephalopathy	25	55.6	10	22.2		
Moderate bilirubin encephalopathy	10	22.2	3	6.7		
Severe bilirubin encephalopathy	0	0.0	0	0.0		

^**^Highly statistically significant at *P* ≤ 0.001

**Fig. 4:**
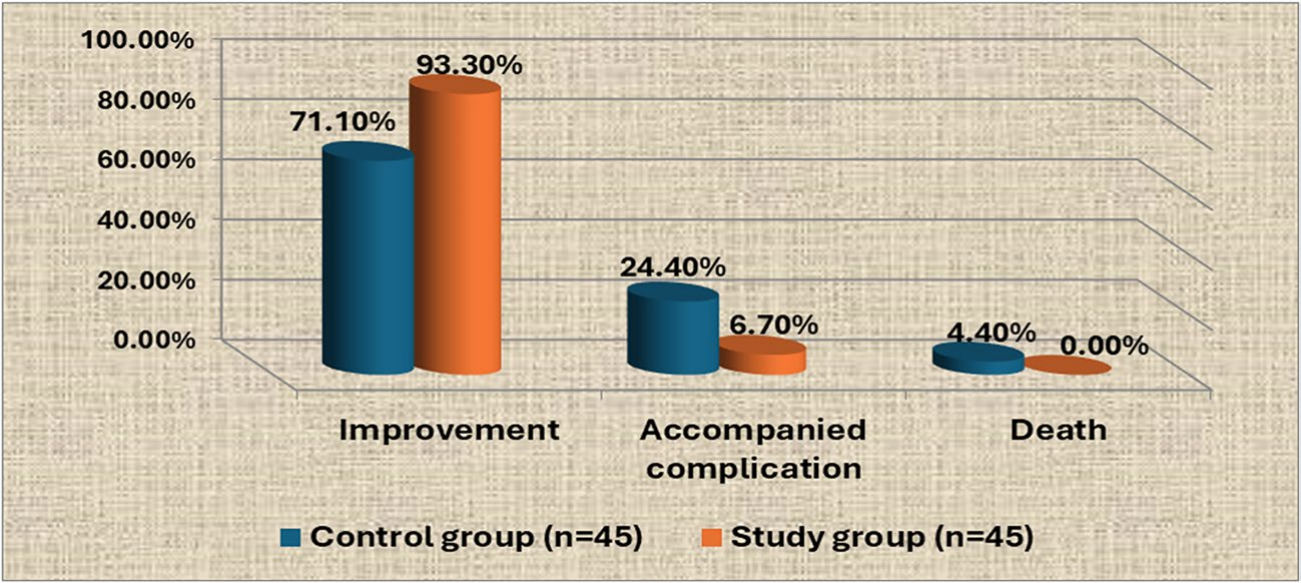
Health outcomes of neonates in the control and study group immediately after discharge from bilisphere phototherapy (*n* = 90)

Figure 4 shows that the majority (93.3%) of neonates in the study group have improvement immediately after discharge from bilisphere phototherapy compared to less than three-quarters (71.1%) in the control group. Furthermore, the vast minority (6.7%) of neonates in the study group have accompanied complication immediately after discharge from bilisphere phototherapy compared to slightly more than one-fifth (24.4%) in the control group.

Table 6 shows that there are a positive correlation between nurses’ total knowledge and total practice regarding bilisphere phototherapy at pre-/post-education program implementation.

**Table 6:** Correlation between the nurses’ total knowledge and total practice regarding Bilisphere phototherapy pre/post Education Program Implementation (*n* = 60)

**Variables**	**Total practice**			
	**Pre- education program implementation (*n* = 60)**		**Post- education program implementation (*n* = 60)**	
	***r***	***p*-value**	***r***	***p*-value**
**Total knowledge**	0.886	0.000**	0.045	0.735

^**^Correlation is significant at the 0.01 level (2-tailed)

Table 7 shows that there are a positive correlation between neonates’ outcomes in the study group and nurses’ total knowledge and total practice regarding bilisphere phototherapy at post-education program implementation with a high statistical significant difference between neonatal outcomes and nurses’ practice regarding skin characteristics and total score of bilirubin-induced neurological dysfunction (*P* ≤ 0.001).

**Table 7:** Correlation between neonates’ outcomes in the study group and nurses’ total knowledge and practice post education program implementation

**Neonates’ outcomes**	**Nurses’ total knowledge**		**Nurses’ total practice**	
	***r***	***p*-value**	***r***	***p*-value**
Vital signs	0.185	0.538	0.416	0.011*
Intended range of lab investigations	0.073	0.651	0.021	0.892
Feeding patterns	0.049	0.746	0.045	0.771
Skin characteristics	0.314	0.057*	0.730	0.001**
Urine characteristics	0.011	0.945	0.019	0.902
Stool characteristics	0.105	0.492	0.019	0.902
Side effects of bilisphere phototherapy	0.048	0.755	0.734	0.003*
Exchange transfusion	0.133	0.384	0.081	0.599
Total score of bilirubin-induced neurological dysfunction	0.518	0.009*	0.483	0.001**

^**^Correlation is highly significant at the 0.01 level (2-tailed). *A statistical significance at *P* ≤ 0.05

## Discussion

Regarding characteristics of the studied nurses (Table 1), it was mentioned that less than half of nurses were in the age group 30 < 40 years, and less than two-thirds of them had technical institute of nursing educational level. This finding was agreed with [29] who carried out a study about “Nurses performance regarding humanized care of newborn with hyperbilirubinemia” and founded that two-fifths (40.0% & 41.4%) of the studied nurses were in the age group 30– < 40 years and had nursing technical institute degree, respectively. The researcher believed that this age category can easily achieve high-quality nursing care and increase the ability to tolerate the working load.

According to characteristics of the studied neonates (Table 2), it was evident that around three-quarters of the control and study groups aged from 1 to 7 days. The current study was matched with [30] who conducted a study entitled “Magnitude and its associated factors of neonatal jaundice among neonates admitted to the neonatal intensive care unit of Dessie Town public hospitals, Amhara region, Ethiopia, 2020: a multicenter cross-sectional study” and declared that more than three-quarters (75.2%) of the studied neonates aged from 1 to 7 days.

More than half of the study group was female, while less than two-thirds of the control group were female neonates. In a study titled “Exploration of the pathogenic factors of neonatal jaundice and the clinical effect of blue phototherapy,” [31] found that over half (52.5%) of the control group and over two-fifths (43.3%) of the study group were female. This result was in line with current findings.

Related to birth weight of neonates, it was found that the control group had mean 3096.6 ± 390.13 g and study group had mean 3016.7 ± 387.32 g. This finding was similar to [32] whose study was entitled “Imaging of nerve injury in neonatal acute bilirubin encephalopathy using 1H-MRS and Glu-CEST techniques” and declared that the mean birth weight of neonates in the control group was 3027 ± 301.1 g and in the study group was 3032 ± 308.3 g. Additionally, the current study was agreed with [33] whose study was entitled “High levels of pathological jaundice in the first 24 h and neonatal hyperbilirubinemia in an epidemiological cohort study on the Thailand-Myanmar border” and found that the mean birth weight of the studied neonates was 3001 ± 420 g.

According to neonates’ type of delivery, it was evident that more than half of both control and study groups were born by cesarean section. This result was agreed with [34] who conducted a study about “Comparison of the effect of foot reflexology and body massage on physiological indicators and bilirubin levels in neonates under phototherapy” and found that the half (50%) of control and study groups were born by cesarean section. On the other hand, the study was disagreed with [35] who conducted a study about “Epidemiology of neonatal jaundice at Punakha District Hospital, Punakha, Bhutan” and revealed that the majority (86.7% & 98.9%) of control and study groups, respectively, were born by normal delivery.

As regards neonates’ type of feeding (Fig. 1), less than three-quarters of the studied neonates in the control and study groups were artificially fed. This finding was agreed with [36], whose study was entitled “Assessment of behavioral and neurological responses of neonates with jaundice undergoing phototherapy” and reported that the majority (90.0%) of the studied neonates were artificially fed. In the same line, the current study consisted with [37], whose study was entitled “Evaluating protocol of management of unconjugated hyperbilirubinemia in neonatal intensive care unit at Qena University Hospital” and showed that more than two-thirds (69.4%) of the studied neonate were artificially fed. From the researcher point of view, this result is due to neonates in NICUs being temporary separated from their mothers plus neonates with pathological jaundice facing effort from breastfeeding.

Regarding neonates’ medical history (Table 3), the current study showed that pathological jaundice caused by RH incompatibility (mother with negative RH) represented less than two-fifths of the study group. This result was in harmony with [38] who conducted a study entitled “Effect of phototherapy on eosinophils count in neonatal hyperbilirubinemia (cross-sectional study)” and found that more than two-fifths (44.5%) of the study neonates had pathological jaundice due to RH incompatibility. From the researcher point of view, these results can be attributed to lack of proper antenatal care and lack of awareness of general population.

Additionally, the current study showed that pathological jaundice caused by sepsis represented less than one-fifth in control and study groups. This result was congruent with [39] who conducted a study entitled “Neonatal jaundice: its determinants among neonates admitted to neonatal intensive care units of Tigray region general hospitals, Northern Ethiopia” and reported that less than one-fifth (18.3%) of the neonates had pathological jaundice due to sepsis. Meanwhile, the current study was disagreed with [40] who conducted a study entitled “Incidence and patterns of neonatal jaundice in tertiary medical facility” and revealed that the minority (4.9%) of the neonate had pathological jaundice due to sepsis. From the researcher point of view, lack of hygiene during and after delivery, poor cord care, and unhygienic newborn care practices may be the major factors in acquisition of neonatal infections and sepsis which caused pathological jaundice.

According to bilirubin level on admission before the use of bilisphere phototherapy, the current study showed that less than two-thirds of control and study groups reached 10 < 20 mg/dL. This finding was matched with [41] who conducted a study entitled “Effectiveness of conventional phototherapy, intensive phototherapy and exchange transfusion in treating neonatal jaundice at Fatima Al-Zahra Hospital for maternity and children in Baghdad” and found that the majority of the studied neonates (83.6%) in the bilirubin level reached 10–19 mg/dL before the use of bilisphere phototherapy.

Regarding studied subjects blood groups, less than one-third and more than one-third of mothers had (O) blood group in control and study groups. In addition, none and the minority of neonates had (O) blood group in control and study groups which explained the result of less than one-third of control and study groups with ABO incompatibility. This result was not matched with [42] who found that around one-third (39.7% & 31.4%) of mothers and neonates, respectively, had (O) blood group and explained the minority (8.3%) of neonates had pathological jaundice due to ABO incompatibility. From the researcher point of view, this result proved the fact of ABO incompatibility which caused by mother with (O) blood group and had neonate with other blood group: A, B, or AB.

As regards studied subjects RH, more than one-third of mothers had negative RH in control and study groups. Additionally, all neonates in control and study groups had positive RH which explained the result of more than one-third of control and study groups with RH incompatibility. This result was not matched with [42] who carried out a study entitled “Assessment of neonatal nurses’ performance regarding early detection of neurological dysfunction among neonates having hyperbilirubinemia” and reported that less than two-thirds (63%) of mothers had negative RH and around two-thirds (66%) of neonates had positive RH which explained less than two-thirds (63%) had pathological jaundice due to RH incompatibility. From the researcher point of view, this result proved the fact of RH incompatibility which caused by mother with negative RH and had neonate with positive RH.

The present study found that most of the nurses under investigation had adequate knowledge about pathological jaundice and bilisphere phototherapy, following the implementation of the education program, based on their overall knowledge of these topics (Fig. 2). This result was consistent with a study by [43] titled “Enhancing neonatal nurses’ performance regarding early detection of neurological dysfunction among neonates having hyperbilirubinemia.” The study found that less than three-quarters (70%) of the nurses studied knew enough about pathological jaundice and how to manage it after implementing an education program. Furthermore, the current study indicated that most nurses (86%) had sufficient knowledge after the intervention, which is in agreement with [44]. This result highlights the significance of conducting a nursing education program because, from the perspective of the researcher, nurses with sufficient knowledge may be able to inform the caregivers and assist in debunking some myths, sociocultural beliefs, and misconceptions regarding neonatal pathological jaundice and bilisphere phototherapy.

Regarding total score of nurses’ practice regarding care for neonates undergoing bilisphere phototherapy (Fig. 3), the current study revealed that the majority of the studied nurses had competent level of total practice, post-education program implementation compared to more than two-thirds pre-education program implementation. This finding was agreed with [45] who conducted a study entitled “Effect of applying aluminum foil reflector during phototherapy combined with nursing care on neonatal hyperbilirubinemia” and found that the majority of the studied nurses (88.3%) had competent level of practice post-intervention program implementation. On the other hand, the current study was not matched with Galala et al. (2023) and declared that more than two-thirds (68.3%) of the studied nurses had incompetent level of total practice pre-program implementation. Additionally, the current study was unharmonious with [46] and showed that more than three-quarters (76.2%) of the studied nurses had incompetent level of total practice pre-program implementation. From the researcher point of view, this finding could be attributed to the absence of continuing educational and training programs at NICU regarding bilisphere phototherapy in different hospitals. Meanwhile, Benha Specialized Pediatric Hospital emphasized training program to nurses towards different diseases.

According to neonates’ medical assessment (Table 4), the current study revealed that the intended range of total serum bilirubin test was achieved in more than two-thirds of the neonates’ studied group. This result was matched with [47] who conducted a study entitled “Intensive phototherapy as the initial management of severe hyperbilirubinemia in neonates: A literature review” and reported that less than two-thirds (64%) of the neonates’ studied group had better value of bilirubin after intensive phototherapy. From the researcher point of view, bilisphere phototherapy had an effective role to control bilirubin level immediately after exposure.

The study and control groups showed a highly statistically significant difference in bilisphere phototherapy side effects (*P* ≤ 0.001). This outcome was consistent with a study by [48] titled “Conventional intensive versus LED intensive phototherapy oxidative stress burden in neonatal hyperbilirubinemia of hemolytic origin” which found that the bilisphere phototherapy group experienced significantly higher rates of skin rash, dehydration, and hyperthermia (*P* = 0.02). From the perspective of the researcher, it is crucial to remember that phototherapy has saved many lives and avoided several cases of serious brain injuries, despite all of its possible hazards and adverse consequences.

As regards neonates exchange transfusion, the current study revealed that the minority of the study group need exchange transfusion after use of bilisphere phototherapy. This result was matched with [49] who founded a study entitled “Intensive 360°(Capsule) versus Conventional Phototherapy in Neonatal Jaundice” and reported that the minority (10.4%) of neonates need exchange transfusion after use capsule phototherapy. Additionally, the current study was congruent with [50] whose study was entitled “Neonatal jaundice: magnitude of the problem in Cairo University's neonatal intensive Care unit as a referral center” and found that the minority (six cases 0.7%) of neonates used intensive phototherapy and exchange transfusion together. From the researcher point of view, this result confirmed the recommendations of the American Academy of Pediatrics (2004) which showed performing exchange transfusion for full-term newborns at least 4 days of age if their TSB level is 25 mg/dL or more and does not decrease sufficiently with phototherapy alone.

According to neonates’ neurological status and the severity of pathological jaundice (Table 5), the current study illustrated that more than one-fifth had mild bilirubin encephalopathy, and the minority had moderate bilirubin encephalopathy of neonates’ study group. This result was in the same line with [50] who found that the minority (2.9%) of neonates’ study group had moderate bilirubin encephalopathy. On the other hand, the current study was inconsistent with [51] who conducted a study entitled “Impact of intensive phototherapy on neurological state of neonates with severe hyperbilirubinemia” and found that after intensive phototherapy, neonates with mild and moderate BIND showed 97.5% and neonates with severe BIND score had persistent evidence of BE (2.5%). From the researcher point of view, BIND score is an important tool to assess neurological status of neonates with pathological jaundice, so early neonates management with bilisphere phototherapy protect neonates from neurological complications and BE.

Regarding health outcomes of neonates immediately after discharge from bilisphere phototherapy (Fig. 4), the current study revealed that the majority of neonates in the study group had improvement immediately after discharge from bilisphere phototherapy compared to less than three-quarters in the control group. This result was congruent with [41] whose study was entitled “Effectiveness of conventional phototherapy, intensive phototherapy and exchange transfusion in treating neonatal jaundice at Fatima Al-Zahra Hospital for maternity and children in Baghdad” and found that the majority (92%) of neonates had successful improvement after intensive phototherapy. Additionally, the current study was supported with [52] who conducted a study entitled “Outcome of neonatal jaundice at Fatima Al-Zahra Hospital for maternity and children in Baghdad” and showed that the majority (99.3%) of the neonates had improvement immediately after discharge from bilisphere phototherapy. On the other hand, the current study was inconsistent with [53] who conducted a study entitled “Predictors of neonatal hyperbilirubinemia's outcome and their relationships to the etiology” and reported that more than three-quarters (75.8%) of the neonates had improvement immediately after discharge from bilisphere phototherapy. From the researcher point of view, bilisphere phototherapy is an important device which had positive outcomes in pediatrics which improve neonates’ health status, but its effect depends on provided effective nursing practice during therapy.

Regarding the correlation between the nurses’ overall knowledge and practice of bilisphere phototherapy (Table 6), the current study showed that both before and after the implementation of the education program, there was a positive correlation between the nurses’ total knowledge and practice of bilisphere phototherapy. This finding was consistent with [44], who discovered a very statistically significant association (*r* = 0.39) between the nurses’ post-intervention total knowledge and practices regarding neonatal hyperbilirubinemia. Similarly, [54] conducted a study titled “A study to assess the effectiveness of protocol on care of newborn in phototherapy on knowledge and practice among nurses at selected hospitals in South India” and found a strong correlation (*r* = 0.80) between nurses’ overall mean improvement level of knowledge and practices regarding newborn care in phototherapy.

Regarding correlation between neonates’ outcomes in the study group and nurses’ total knowledge and practice, post-education program implementation (Table 7), the current study represented that there were a positive correlation between neonates’ outcomes in the study group and nurses’ total knowledge and total practice regarding bilisphere phototherapy at post-education program implementation. This result was congruous with [42] who reflected that there were highly statistical significant positive correlations between nurses’ total knowledge, total practices, and neonates’ outcomes which it was found that the higher the level of nursing practices, the fewer the frequency of side effects of phototherapy. From the researcher point of view, equipping nurses with knowledge and practice and maintaining updates with professional training provide high quality of neonate care.

### Limitations of the study:

A few limitations applied to this study: there are significant regional differences in the pattern of etiological variables for neonatal pathological jaundice. Our findings may not be indicative of the trend in other regions as a result. Only English-language publications were included in our inquiry; thus, we probably missed some important studies, especially those pertaining to Chinese, Turkish, and traditional medicine.

## Conclusion

The research hypotheses were validated based on the results of the current investigation. Compared to before the education program was implemented, the improved nurse’s performance results on improved bilirubin-induced neurological dysfunction score of neonates. Additionally, after the program was put into place, there was a good association between the performance of nurses and the outcomes of neonates.

### Recommendation

According to the real needs of nurses, periodic in-service education programs should be created and put into place in neonatal intensive care units to enhance nurses’ skills and knowledge. These programs should include audiovisual conferences and films regarding practical procedures for nurses. Educate moms of the dangers of severe neonatal jaundice during prenatal care, particularly if she has blood group O or Rh negative. Additionally, if a newborn exhibits jaundice within the first 24 h of life, counsel parents to seek immediate medical attention from a qualified healthcare provider. To prevent post-phototherapy rebound, teach parents to monitor newborns with hemolytic jaundice.

## Data Availability

The data that support the findings of this study are available on request from the corresponding author.
